# A Spherically-Shaped PZT Thin Film Ultrasonic Transducer with an Acoustic Impedance Gradient Matching Layer Based on a Micromachined Periodically Structured Flexible Substrate

**DOI:** 10.3390/s131013543

**Published:** 2013-10-09

**Authors:** Guo-Hua Feng, Wei-Fan Liu

**Affiliations:** 1 Department of Mechanical Engineering, National Chung Cheng University, Chiayi 621, Taiwan; E-Mail: answer0131@hotmail.com; 2 Advanced Institute of Manufacturing with High-Tech Innovations, National Chung Cheng University, Chiayi 621, Taiwan

**Keywords:** PZT film, ultrasonic transducer, micromachining, acoustic impedance

## Abstract

This paper presents the microfabrication of an acoustic impedance gradient matching layer on a spherically-shaped piezoelectric ultrasonic transducer. The acoustic matching layer can be designed to achieve higher acoustic energy transmission and operating bandwidth. Also included in this paper are a theoretical analysis of the device design and a micromachining technique to produce the novel transducer. Based on a design of a lead titanium zirconium (PZT) micropillar array, the constructed gradient acoustic matching layer has much better acoustic transmission efficiency within a 20–50 MHz operation range compared to a matching layer with a conventional quarter-wavelength thickness Parylene deposition. To construct the transducer, periodic microcavities are built on a flexible copper sheet, and then the sheet forms a designed curvature with a ball shaping. After PZT slurry deposition, the constructed PZT micropillar array is released onto a curved thin PZT layer. Following Parylene conformal coating on the processed PZT micropillars, the PZT micropillars and the surrounding Parylene comprise a matching layer with gradient acoustic impedance. By using the proposed technique, the fabricated transducer achieves a center frequency of 26 MHz and a −6 dB bandwidth of approximately 65%.

## 1. Introduction

High-frequency (>20 MHz) focused ultrasonic transducers appear in many applications, including high-resolution medical imaging, therapeutic treatment, and nondestructive material testing [1–4]. However, most transducers created by using the piezoelectric micromachined ultrasonic transducer (pMUT) or capacitive micromachined ultrasound transducer (cMUT) schemes are based on a planar substrate, which typically limits their geometric designs.

Highly sensitive broadband ultrasonic transducers require efficient transfer of acoustic energy between the active piezoelectric material and the propagating medium. A conventional acoustic impedance matching layer can be made with the following materials: (a) suspended particles surrounded by a polymer matrix prepared separately and then bonded to the piezoelectric ceramic layer; (b) a composite of solid and polymer filler materials with specific acoustic impedance. These composites are also made separately and must bond to the active piezoelectric ceramic layer; (c) a piezocomposite (e.g., 1–3 composites) made of a piezoelectric ceramic and a passive conformal polymer. The acoustic impedance of the active piezoelectric layer can be set at a specific value for acoustic matching purposes; (d) an integrated acoustic impedance matching layer with a series of microgrooves at the surface of the bulk piezoelectric ceramic. The depth of the grooves determines the thickness of the integrated impedance matching layer, and a conformal filler material is used to fill the grooves [5–9].

However, most bulk piezoelectric ceramic transducers require appropriate acoustic matching layers to enhance acoustic energy transfers. This can be difficult to implement when using a piezoelectric thin film to make high-frequency ultrasonic transducers. In addition, this type of thin film makes it difficult to build appropriate acoustic matching layers on spherically-shaped ultrasonic transducers with the designed curvatures.

In this paper, we propose a novel pMUT fabrication method. High-performance lead titanium zirconium (PZT) film can be deposited on a flexible substrate with arbitrary curvature to form spherically-shaped transducers, and microstructured PZT bumps on the PZT film and the Parylene material can form an acoustic impedance gradient matching layer to reduce the acoustic impedance, yet increase the coupling coefficient and the operating bandwidth of the ultrasonic transducer.

## 2. Consideration of Device Design and Fabrication Process

**Figure 1.**
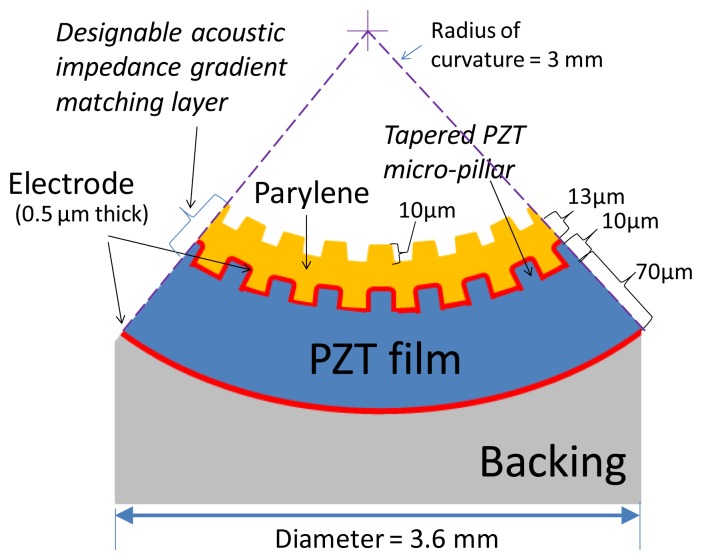
Illustration of the configuration of the cross-sectional view of proposed spherically-shaped ultrasonic transducer with an acoustic impedance gradient matching layer.

Figure 1 shows the configuration of the proposed ultrasonic transducer. The piezoelectric PZT thin film on the ultrasonic device can be fabricated with an arbitrary curvature, and the acoustic impedance of the matching layer on top of the PZT film can be fabricated according to design requirements. Because the proposed fabrication process of the acoustic impedance gradient matching layer is novel, we will present the challenges and solutions for producing the ultrasonic device. This study also presents a discussion of the design parameters and transmission efficiency of the acoustic matching layer.

Three major considerations are involved in fabricating a transducer: (a) A curvature tunable, spherical geometry is needed as a substrate for a focused ultrasonic beam design; (b) A piezoelectric ceramic material serves as the ultrasound generation material. This type of material usually has a higher electromechanical coupling coefficient and piezoelectric constant than a piezoelectric polymer material. A high annealing temperature (e.g., >500 °C) is also required to achieve high film quality; (c) An acoustic impedance gradient matching layer must be fabricated and easily integrated on the front of the deposited piezoelectric material.

To achieve these design requirements, a metal sheet has been used as the substrate for PZT film deposition [10,11]. This substrate is then micromachined to form periodic microcavities for the subsequent fabrication of the acoustic impedance gradient matching layer. Because of its ductility, a metal sheet can be easily shaped by mechanical force. An embossing scheme is used to form the required curvature. Steel balls with an identically designed diameter are aligned and pressed into the metal sheet at specific locations, forming uniform microcavities spread on the curved surface of the forged metal sheet. This step is followed by PZT film deposition. A sol-gel spray method is used to deposit the PZT layer by layer on the processed metal substrate. A high annealing temperature, typically above 500 °C, is required to achieve a high-quality piezoelectric film; the substrate must be sustainable in this environment. Metal is then deposited to form the electrode, and tungsten-adulterated epoxy is applied as the backing layer. The shaped metal is then etched away so that the curved piezoelectric thin film is supported by the epoxy backing material, and the periodic microstructured piezoelectric pillars appear on top of the curved piezoelectric film. Additional metal is deposited on the top electrode of the ultrasonic transducer. The active ultrasound generator is then complete, and an acoustic matching layer is necessary to improve the acoustic energy transmission for biomedical imaging and other ultrasonic applications.

Conformal Parylene was deposited on top of the piezoelectric film with a micropillar array to create the acoustic impedance gradient matching layer. Because the cross-sectional area of the micropillars monotonically decreases from the piezoelectric film outward, the mixed structure of the Parylene layer and the PZT micropillars forms a varied acoustic impedance matching layer. The dimensions of the micropillars and thickness of the deposited Parylene could be critical parameters in adjusting the acoustic impedance of the composite layer.

**Figure 2.**
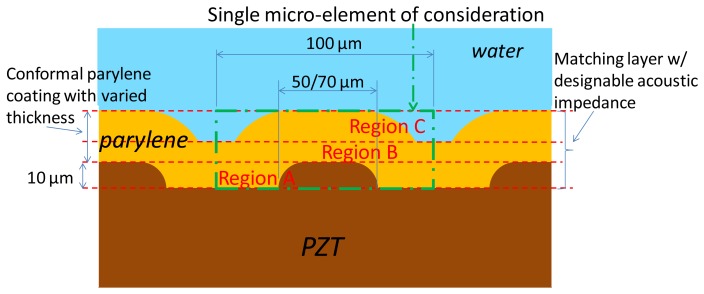
Illustration of the acoustic matching layer design containing the fabricated composite consisting of PZT micropillars and variable thickness Parylene to form Regions A, B, and C.

To evaluate the transmission efficiency and identify superior acoustic energy coupling designs, we simplified the concave geometry effect and considered the micropillars on the flat piezoelectric film with a conformal coating of Parylene. Because of the periodic property of these micropillars, a single microelement within the acoustic matching layer contains a micropillar in the center region (Figure 2). The element is 100 μm long. The largest and smallest diameters of the pillar are 70 μm and 50 μm, respectively. This is because of the ideal isotropic chemical etching of the metal substrate, based on 50-μm and 30-μm circular opening patterns, respectively, and 10-μm deep etching for both cases. In this study, we focused on the acoustic transmission efficiency of varying Parylene thicknesses when the deposited Parylene is thicker than the height of the piezoelectric pillar.

Unlike common acoustic matching layer designs, which are based on a quarter-wavelength concept, the transmission coefficient of the designed acoustic impedance gradient matching layer is difficult to calculate. Here, we used the integration concept by slicing the acoustic matching layer into individual thin layers, each of which has a different acoustic property. The varied acoustic parameters of the virtual sliced thin layers are then derived, and their transmission coefficients are calculated. Finally, the numerical results of the designed acoustic impedance gradient matching layer and the analytical results of a simple quarter-wavelength planar acoustic matching layer are presented and compared.

### 2.1. Equivalent Acoustic Impedances of Virtual Sliced Thin Layers

Because the PZT/Parylene volume fractions in Region A of the designed acoustic matching composite layer are varied along the axis perpendicular to the active piezoelectric section, we sliced Region A into several virtual thin layers with different acoustic properties. The equivalent sound velocity *c_eq_* and acoustic impedance *Z_eq_* of each thin layer within region A (Figure 2) can be represented as follows:

(1) $$c_{eq}=\sqrt{\kappa_{eq}/\rho_{eq}}$$

(2) $$Z_{eq}=\sqrt{\kappa_{eq}\cdot \rho_{eq}}$$

where *ĸ_eq_* and *ρ_eq_* are the equivalent bulk modulus and density, respectively. The equivalent density can be derived from the fractional composition of different materials as follows:

(3) $$\rho_{eq}=\rho_{\text{PZT}}\cdot V_{\text{PZT}}+\rho_{\text{parylene}}\cdot (1-V_{\text{PZT}})$$

The term *V_PZT_* represents the volume fraction of PZT within the thin composite layer of interest. For the first-order approximation, consider the individual PZT micropillars and Parylene layers that form the composite to be isotropic. The equivalent bulk modulus can be estimated as:

(4) $$\kappa_{eq}=\frac{(\kappa_{\text{PZT}}+G_{\text{parylene}})\kappa_{\text{parylene}}+(\kappa_{\text{pzt}}-\kappa_{\text{parylene}})G_{\text{parylene}}V_{\text{PZT}}}{(\kappa_{\text{PZT}}+G_{\text{parylene}})-(\kappa_{\text{pzt}}-\kappa_{\text{parylene}})V_{\text{PZT}}}$$

(5) $$\kappa_{\text{PZT}}=\frac{Y_{\text{PZT}}}{3(1-2\nu_{\text{PZT}})}\kappa_{\text{parylene}}=\frac{Y_{\text{parylene}}}{3(1-2\nu_{\text{parylene}})}G_{\text{parylene}}=\frac{Y_{\text{parylene}}}{2(1+\nu_{\text{parylene}})}$$

where *ĸ_PZT_* and *ĸ_parylene_* are the bulk moduli of PZT and Parylene, respectively. The term *G_pzrylene_* represents the shear modulus of Parylene. The terms *Y_PZT_* and *Y_parylene_* are the Young's moduli of PZT and Parylene, respectively. These formulas are based on an analytical method using a self-consistent field micromodel that provides a better estimation of composite properties than the mechanics of materials approach. This model assumes the composite to be a concentric cylinder with a transversely isotropic matrix and permits formulations based on the theory of elasticity to determine the stress and strain variations in a realistic manner [12].

Region B consists of Parylene, and its acoustic property can be easily determined. Region C of the designed matching layer consists of wavy-shaped Parylene microstructures surrounded by water (Figure 2). The equivalent sound velocity *c_eq_* and acoustic impedance *Z_eq_* of each thin layer within this region can be simply expressed by their volume fractions as follows:

(6) $$c_{eq}=\sqrt{\left[\kappa_{\text{parylene}}V_{\text{parylene}}+\kappa_{\text{water}}(1-V_{\text{parylene}})]/[\rho_{\text{parylene}}V_{\text{parylene}}+\rho_{\text{water}}(1-V_{\text{parylene}})\right]}$$

(7) $$Z_{eq}=\sqrt{\left[\kappa_{\text{parylene}}V_{\text{parylene}}+\kappa_{\text{water}}(1-V_{\text{parylene}})]\cdot [\rho_{\text{parylene}}V_{\text{parylene}}+\rho_{\text{water}}(1-V_{\text{parylene}})\right]}$$

where *V_parylene_* represents the volume fraction of Parylene within the thin layer of interest.

The physical properties of materials are listed in Table 1 for our following calculations.

### 2.2. Acoustic Transmission Coefficient Calculation

This study considers the propagation of an acoustic wave in the designed matching layer as a plane wave for a first-order approximation. For a given incident acoustic wave:

(8) $$p_{i}=A_{0}\cdot e^{j(\omega t-k_{0}x)}$$

(9) $$\rho_{0}\frac{\partial {\vec{u}}_{i}}{\partial t}=-\nabla p_{i}$$

where the terms *p_i_* and *ū_ι_* are the acoustic pressure and particle velocity of the incident wave, respectively. The term *A*_0_ is the pressure amplitude, *ρ*_0_ is the density of the medium, *ω* is the angular frequency, *k* is the wave number, and *x* is the position. The transmitted *p_t,n_* and reflected *p_r,n_* acoustic waves within the nth thin virtual layer can be described as [13]:

(10) $$p_{t,n}=A_{n}\cdot e^{j(\omega t-k_{n}x)}$$

(11) $$p_{r,n}=B_{n}\cdot e^{j(\omega t+k_{n}x)}$$

The boundary conditions of continuity of pressure and continuity of regular velocity must be satisfied for all times at all points on the interface. For the interface at *x* = *d_n_*, we have:

(12) $$A_{n}\cdot e^{-jk_{n}d_{n}}+B_{n}\cdot e^{jk_{n}d_{n}}=A_{n+1}\cdot e^{-jk_{n+1}d_{n}}+B_{n+1}\cdot e^{jk_{n+1}d_{n}}$$

(13) $$\frac{A_{n}}{Z_{n}}\cdot e^{-jk_{n}d_{n}}-\frac{B_{n}}{Z_{n}}\cdot e^{jk_{n}d_{n}}=\frac{A_{n+1}}{Z_{n+1}}\cdot e^{-jk_{n+1}d_{n}}-\frac{B_{n+1}}{Z_{n+1}}\cdot e^{jk_{n+1}d_{n}}$$

Further derivation produces the following relation:

(14) $$\begin{array}{c} \left[\begin{array}{c} A_{n}\\ B_{n} \end{array}\right]=\frac{1}{2Z_{n+1}}\left[\begin{array}{cc} (Z_{n+1}+Z_{n})\cdot e^{-j(k_{n+1}-k_{n})d_{n}} & (Z_{n+1}-Z_{n})\cdot e^{j(k_{n+1}+k_{n})d_{n}}\\ (Z_{n+1}-Z_{n})\cdot e^{-j(k_{n+1}+k_{n})d_{n}} & (Z_{n+1}+Z_{n})\cdot e^{j(k_{n+1}-k_{n})d_{n}} \end{array}\right]\;\left[\begin{array}{c} A_{n+1}\\ B_{n+1} \end{array}\right]\\ =T_{n}\cdot \left[\begin{array}{c} A_{n+1}\\ B_{n+1} \end{array}\right] \end{array}$$

Assuming that the acoustic wave transmits multilayers (from *n* = 0 to *n* = *m* + 1) with various acoustic properties, we can then express the transmission matrix *T* as:

(15) $$T=\prod_{n=0}^{m+1}T_{n}$$

(16) $$\left[\begin{array}{c} A_{0}\\ B_{0} \end{array}\right]=T\cdot \left[\begin{array}{c} A_{m+1}\\ B_{m+1} \end{array}\right]$$

If we consider the last layer (*n* = *m* + 1) as the target medium, which has no reflected wave, the coefficient *B_m_*_+1_ should be zero. The pressure transmission coefficient *T_p_* and intensity transmission coefficient *T_I_* can be determined as:

(17) $$T_{P}=\frac{1}{T(1,1)}$$

(18) $$T_{I}=\frac{Z_{0}}{Z_{m+1}}{\left|\frac{1}{T(1,1)}\right|}^{2}$$

### 2.3. Numerical Results of the Designed Gradient Acoustic Matching Layer and Comparison with Planar Acoustic Matching Layer Design

**Figure 3.**
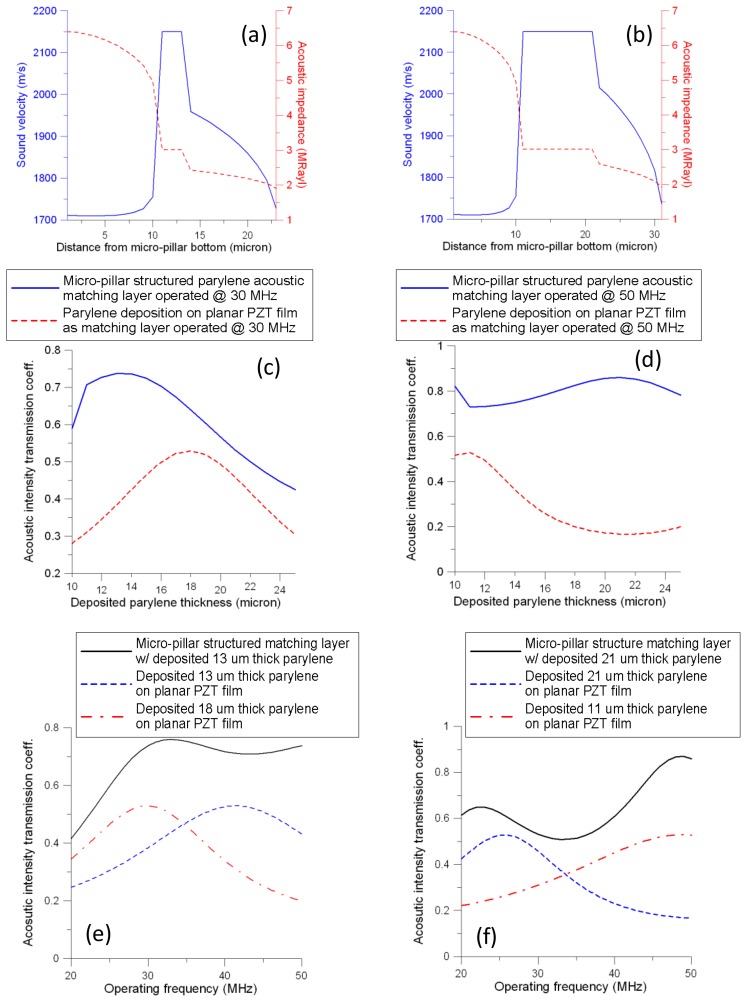
Numerical results: (**a**,**b**) sound velocity/acoustic impedance as the function of the distance from the bottom of designed micropillar outward for two different cases of 13 and 21 μm Parylene deposition, respectively (Figure 2); (**c**,**d**) Comparison of the acoustic intensity transmission coefficients of the PZT micropillar structured and simply planar PZT structured matching layer with varied parylene thicknesses; (**e**,**f**) Frequency responses of the acoustic intensity transmission coefficients based on the given thickness of Parylene deposition.

Figure 3a,b show the numerical results of the equivalent sound velocity and acoustic impedance as a function of the distance from the bottom of the designed micropillar outward for two cases (13-μm and 21-μm Parylene deposition). Both designs display a substantial decrease in the trend of acoustic impedances in Regions A and C (Figure 2). In Region B, the sound velocity and acoustic impedance are constant, but have different spacing intervals for the cases with 13-μm and 21-μm Parylene deposition. For both cases, the largest acoustic transmission intensity coefficient occurs at 30 MHz and 50 MHz, respectively (Figure 3c,d). Various operating frequencies can be used for the proposed micropillar-structured acoustic matching layer design.

The common method directly deposits an acoustic impedance matching layer onto a planar piezoelectric film. The acoustic intensity transmission coefficient can be formulated as follows if Parylene is the matching material [14]:

(19) $$T_{I}=\frac{4}{2+(\frac{Z_{\text{water}}}{Z_{\text{PZT}}}+\frac{Z_{\text{PZT}}}{Z_{\text{water}}})\cos^{2}k_{\text{parylene}}L+(\frac{Z_{\text{parylene}}^{2}}{Z_{\text{PZT}}\cdot Z_{\text{water}}}+\frac{Z_{\text{PZT}}\cdot Z_{\text{water}}}{Z_{\text{parylene}}^{2}})\sin^{2}k_{\text{parylene}}L}$$

Figure 3c,d show a comparison of the acoustic intensity transmission coefficients of the PZT micropillar structure and simple planar PZT matching layer with various Parylene thicknesses. The results in these figures indicate the superiority of the micropillar matching layer. For the same thickness of Parylene deposition, the micropillar matching layer consistently achieves better acoustic intensity transmission performance than a simply planar Parylene matching layer, particularly for the 50 MHz case.

Although the planar Parylene acoustic matching layer can be easily calculated with Equation (19), its limited performance is a major disadvantage. The maximal acoustic intensity transmission coefficients occur at a matching layer equal to one-quarter of the wavelength of the transmitted wave (*i.e.*, 18-μm thick and 11-μm thick Parylene for 30 MHz and 50 MHz operating frequencies, respectively). The coefficients are approximately 0.53 and are dominated by the term $Z_{\text{parylene}}^{2}/(Z_{\text{PZT}}\cdot Z_{\text{water}})+(Z_{\text{PZT}}\cdot Z_{\text{water}})/Z_{\text{parylene}}^{2}$. Compared to the micropillar acoustic matching layers, the maximal coefficients of acoustic intensity transmission are 0.76 and 0.86. These coefficients occur at Parylene deposition thicknesses of 13 μm and 21 μm, respectively, if the operating frequencies are 30 MHz and 50 MHz, respectively.

Figure 3e,f shows the frequency responses of the acoustic intensity transmission coefficients based on the given thickness of Parylene deposition. The proposed micropillar acoustic impedance matching layer demonstrates substantially higher operating bandwidth and efficiency than the matching layer of planar PZT with simple Parylene film deposition.

## 3. Device Fabrication

### 3.1. PZT Precursor Preparation

We used a PZT slurry to deposit the PZT film and micropillars. This slurry consists of precursor and PZT powder. Four chemicals were included in the precursor solution [15–18]: lead acetate trihydrate [Pb(CH_3_COO)_2_·3H_2_O], zirconium *n*-propoxide [Zr(OCH_2_CH_2_CH_3_)_4_], titanium *n*-butoxide [Ti(OC_4_H_9_)_4_], and 2-methoxyethanol (CH_3_OCH_2_CH_2_OH). First, lead acetate trihydrate (13.28 g), zirconium *n*-propoxide (8.18 g), and titanium *n*-butoxide (7.09 g) were separately dissolved in 2-methoxyethanol (86.72, 41.82, 42.91 g) and refluxed at 130 °C for 2 h.

These three solutions were then stored in individual glass bottles for further processing. While the PZT precursor solution was prepared, the three methoxyethanol solutions, stored at room temperature, were decanted into a flask, and lead acetate trihydrate (10 g) was added to the mixed solution to compensate for the evaporation of PbO in the following heating step. The processed solution was then refluxed at 130 °C for 6 h and stirred with a magnetic bar. After cooling to room temperature, the PZT precursor solution (with a mole ratio of lead acetate trihydrate:zirconium *n*-propoxide:titanium *n*-butoxide = 1.05:0.52:0.48) was stored in a bottle in a dry environment.

The PZT powder and glycerol were then added to the solution to increase the thickness of PZT film formation and its piezoelectric properties. The PZT powder had an average particle size of 0.5 μm (Eleceram Technology Co., Taoyuan, Taiwan). Glycerol, which served as the chelating agent, was used to adjust the viscosity of the mixed PZT powder and precursor solution to form a slurry. The ratio of the PZT solution, PZT powder, and glycerol was 10:1:2. An ultrasonic bath was used to ensure efficient mixing for 2 h, and the resulting slurry was then ready for further processing.

### 3.2. Fabrication Process and Results

To form a piezoelectric ultrasonic transducer with a novel micropillar acoustic matching layer, we used a 100-μm thick copper sheet (purity 99%) as the substrate. Because copper is a ductile metal with a high thermal conductivity, and its melting temperature is 1,084 °C, it is a suitable candidate material for the subsequent processes, which involve high-temperature annealing and mechanical shaping. An array of 10-μm deep circular cavities was fabricated on the copper substrate by micromachining. This was performed by a photolithography and wet-etching process. The circular holes of diameter 50 μm are used for the photomask design. Because of the isotropic etching effect of copper, AZ5214 positive photoresist was used to pattern the designed circles. The photomask design should consider the size differences between the plotted holes and etching finished holes. The etching process was performed using a diluted copper etchant (etchant:water = 1:4) for 10 min, and the photoresist was then removed.

**Figure 4.**
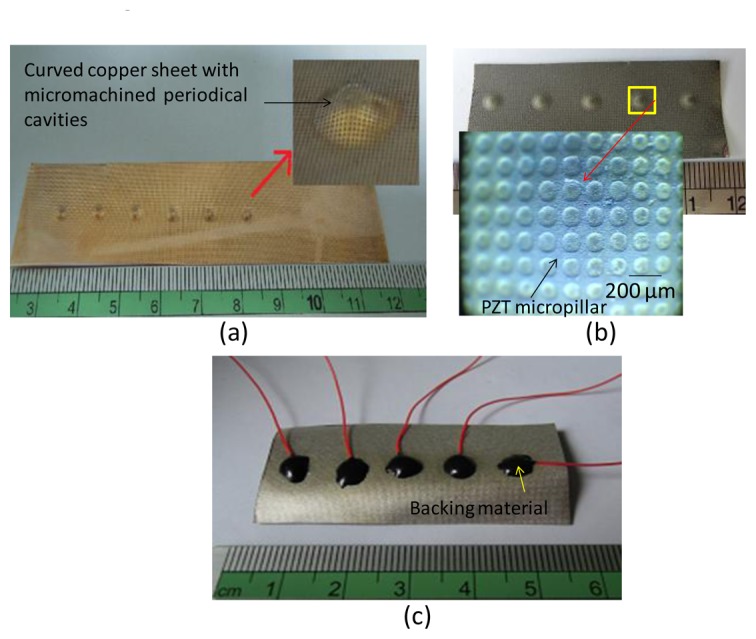
(**a**) A copper sheet was micromachined to form periodic micro-patterned cavities and easily imprinted as designed arbitrary curvatures at specific locations; (**b**) The deposited PZT layer fully covered the microcavities on the shaped copper substrate; (**c**) E-Solder 3021 applied as the backing material.

The copper sheet was subsequently imprinted with a steel ball of diameter 6 mm to produce a designed curvature as the substrate for PZT film deposition. The depth of the ball pressed into the metal is 0.6 mm, and the resulting outer diameter of the active bowl is 3.6 mm. Figure 4a shows the fabrication results. This method can be used to form an array of transducers in a one-step process. An approximately 80-μm thick PZT film was fabricated using the proposed Multiple Spray and Bake followed by Sintering (MSBS) process. The PZT slurry was used to deposit a PZT film. The MSBS process could easily deposit 10-μm to 100-μm thick PZT films.

The details of the MSBS process are described as follows. A spray method was used to deposit PZT film on the shaped copper sheet substrate. During the spray process, the nozzle of the spray gun was placed 30 cm from the copper sheet at a 45° angle to achieve favorable uniformity.

The whole deposition process was divided into several repeated deposition cycles. The repeated cycle number depends on the designed PZT thickness. In this study, four cycles were processed. Each deposition cycle involved spraying 20 times. The first deposition cycle consisted of 2 min of soft baking (150 °C) and 1 min of cooling (room temperature) in an oven. After repeating the spray-soft bake-cooling procedure 20 times, the device was placed in an oven for high-temperature treatment (*i.e.*, a hard bake). The temperature was initially set at 350 °C, increasing from 150 °C at a rate of 10 °C/min. A temperature of 350 °C was maintained for 10 min before cooling to room temperature. The first deposition cycle was then complete. The second, third, and fourth deposition cycles were then repeated as for the first cycle. After finishing the fourth deposition cycle, the processed copper sheet substrate was placed in an oven for annealing treatment. The annealing temperature (e.g., 600 °C) was reached by 40 °C/min and maintained at the annealing temperature for 30 min. The heat source was then extinguished, and the wafer remained in the oven to cool for 5 h. Deposition was complete at this time, and the PZT slurry fully covered the microcavities on the shaped copper substrate (Figure 4b).

**Figure 5.**
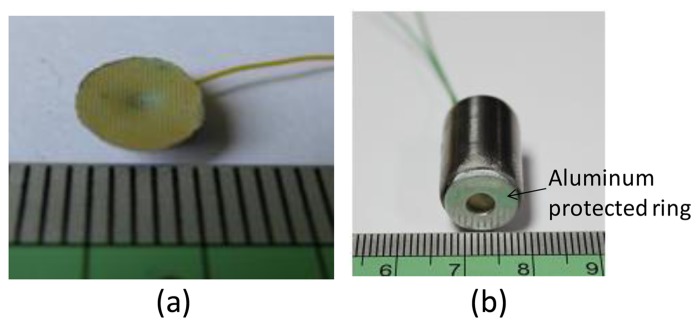
(**a**) Finished ultrasonic transducer with an acoustic impedance gradient matching layer; (**b**) The fabricated transducer packaged in an aluminum tube.

To make ultrasonic transducers, a bottom aluminum electrode of 0.5 μm was sputtered onto the fabricated PZT film with a shadow mask. After connecting wires to the top and bottom electrodes, E-Solder 3021 was applied as the backing material (Figure 4c). The copper substrate of the device was removed by copper etchant, and uniform PZT micropillars appeared on the surface of the curved PZT film. This tapered micropillar array allows us to structure an acoustic impedance gradient matching layer. A 0.5-μm thick aluminum layer was then sputtered as the top electrode. A 13-μm thick Parylene layer, according to the aforementioned calculation for 30 MHz operating frequency, was then conformally deposited on the processed device (Figure 5a). The focusing inner region is active, and the flat outer ring portion is not active. To separate these two regions, we made a shadow mask consisting of a copper sheet with a designed opening to define the active region. Then this shadow mask was put in front of the transducer, and a 0.5-μm thick aluminum layer was sputtered as the top electrode. The fabricated transducer was packaged in an aluminum tube (Figure 5b). The silver ring on the front of the packaged transducer is a protective ring. Polarization was performed by immersing the device in a 120 °C silicone oil environment and applying 70 V between the top and bottom electrodes for 30 min with an electric field setting of 1 kV/mm.

## 4. Device Characterization and Discussion

### 4.1. Characterization of the Ball-Imprinted Copper Sheet with Microcavities

**Figure 6.**
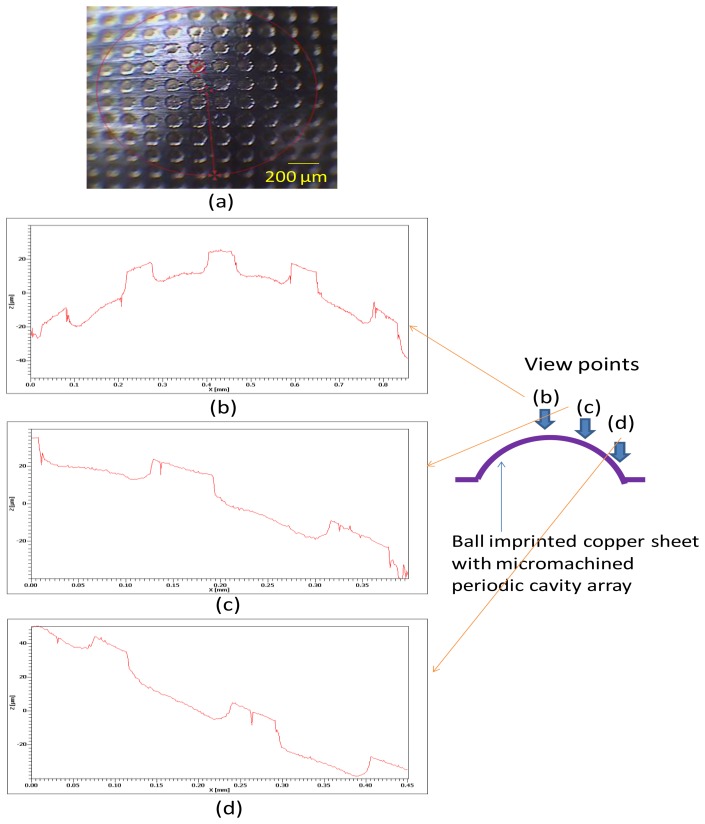
(**a**) Microscopic image of the ball-imprinted copper sheet with periodic micromachined cavities; the image shows the highest region of the sheet; (**b**–**d**) Profiles of the shaped copper sheet at the vertex region, the middle zone, and the bottom area, taken from the white light interferometer.

Figure 6a shows a localized microscopic image of the ball-imprinted copper sheet with periodic micromachined cavities; this image shows the highest region of the sheet. In this case, steel balls with a diameter of 6 mm were used to emboss the flat side of the copper sheet. Figure 6 show the profiles of the shaped copper sheet at the vertex, middle, and bottom areas, as recorded by a white-light interferometer. The periodic plateau and indentation regions were produced with step distances of 15 μm in the vertical direction. Each formed indentation exhibits a gradually decreasing inward-opening area caused by isotropic etching, confirming the proposed model. The surface curvature of the entire cavity exhibits a decreasing trend from the vertex to the bottom region. This is because the ball starts to press the copper sheet at the vertex location and arrives at the middle zone of the cavity during the imprinting process. The bottom area is therefore stretched to a nearly flat surface.

If we examine the spacing between the corners of neighboring plateaus, the distances projected on the horizontal surface (x-axis) maintain the same value (approximately 120 μm; for different locations. A noteworthy phenomenon is that the originally symmetrical indentation becomes distorted after the imprinting process. That is, the two curvatures formed by the two sidewalls and bottom of an indentation are different depending on their locations at various latitudes of the shaped cavity.

### 4.2. X-Ray Diffraction Characterization of the Fabricated PZT Film

**Figure 7.**
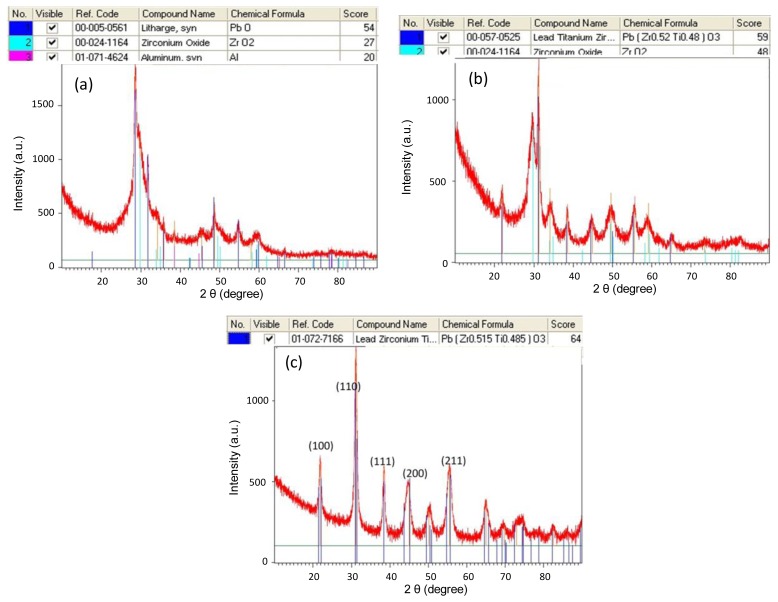
XRD spectra of deposited PZT film for different annealing temperatures: (**a**) 500 °C; (**b**) 525 °C; (**c**) 600 °C.

We used X-ray diffraction (xRD) to examine the crystallization and related phase of the fabricated PZT film. The characterization was performed on a PANalytical X'Pert Pro X-ray Diffraction System from 5° to 90° (2θ) with a scanning speed of 4°/min and a step size of 0.02° employing Cu Kα radiation (λ = 1.5406;. Figure 7 shows the resulting XRD spectra for different annealing temperatures. The associated crystal orientations of PZT for (100), (110), (111), (200), and (211) approximately correspond to the 2θ angles of 22°, 31°, 38°, 44°, and 55°. For an annealing temperature of 500 °C, the perovskite structure of Pb(Zr, Ti)O3 did not form. The major peak shows that PbO is dominant in the processed film. When the annealing temperature increases to 525 °C, Pb(Zr_0.52_, Ti_0.48_)O_3_ exhibits a perovskite structure. A strong peak of nearly 31° shows a good (110) orientation, but ZrO2 composition also exists. When the annealing temperature reaches 600 °C, all the measured peaks indicate a good perovskite PZT formation.

### 4.3. Experimental Setup and Results of Pulse-Echo Measurement

**Figure 8.**
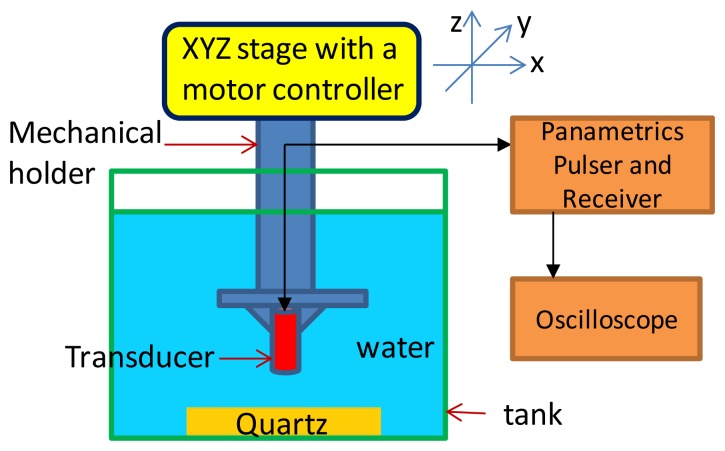
Illustration of the pulse-echo experimental setup.

**Figure 9.**
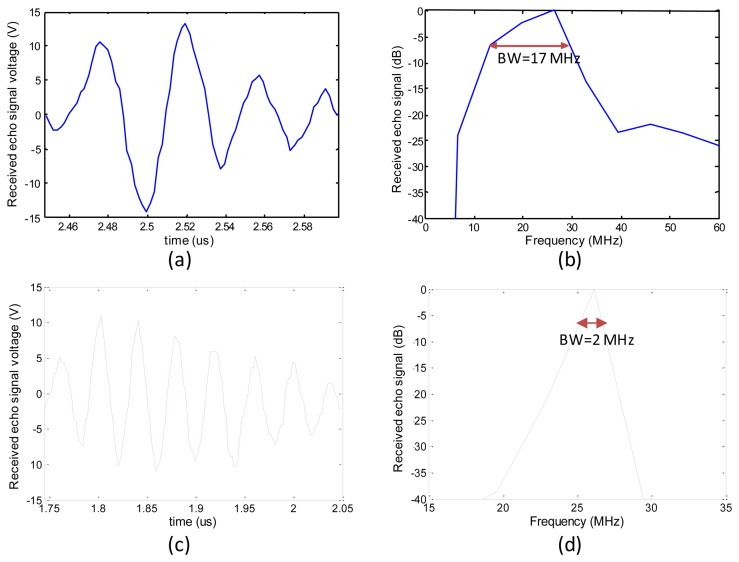
Pulse-echo response of the fabricated ultrasonic transducer: (**a**) Fabricated curved transducer with gradient acoustic impedance matching layer; (**b**) Fourier transform spectrum of (a); (**c**) The same sized spherically-shaped transducer uses a 18 μm thick parylene layer as an acoustic matching layer for comparison; (**d**) Fourier transform spectrum of (c).

The packaged ultrasonic transducer was also characterized in a pulse-echo experiment. Figure 8 shows the experimental setup. The transducer was anchored onto a platform of an x-y-z three-axis moving stage (1 μm displacement resolution for the three axes). The quartz reflector was placed 2 mm from the transducer. The echo signal from the receiver was monitored and recorded on a 500 MHz bandwidth LeCroy LT342 digital oscilloscope. The acquired signal was used to find the frequency spectrum by Matlab software. The pulser/receiver had the following settings: pulse repetition rate of 1 kHz, input energy of 2 μJ, damping of 50 Ω, gain of 39 dB, high pass filter of 1 kHz, and low pass filter of 75 MHz. Figure 9 shows the time domain and frequency domain responses of the transducer. The peak-to-peak voltage is about 28 V for the transducer with a gradient acoustic impedance matching layer. The FFT spectrum analysis indicates the maximum frequency is at 26 MHz. The center frequency is in the range of 21 MHz and a −6 dB bandwidth of 65%.

We also fabricated the same sized spherical PZT thin film transducer with an 18 μm thick Parylene film as the acoustic matching layer to compare the transducer with the acoustic impedance gradient matching layer. Following the same aforementioned pulse-echo experimental procedure, the results for the compared transducer are shown in Figure 9c,d. The results show the peak-to-peak voltage of the echo signal for the compared transducer is about 21 V. The center frequency is 26 MHz and a −6 dB bandwidth of 8%.

To further evaluate the radiation characteristics of the transducers with and without the acoustic impedance gradient matching effect, we have the discussion below. Assume the PZT films of both transducers had the identical piezoelectric quality. The acoustic intensities generated by these two transducers were the same during the electrical pulse triggers. The produced acoustic wave traveled through the matching layer and water, and then reflected from the quartz reflector. It is followed by passing through the water and matching layer and finally arrived at the PZT film. The distances between the reflectors and the transducers for two cases were equal. The intensity of the acoustic wave transmitting through the reflector was negligible due to larger acoustic impedance mismatch between the quartz reflector and water. Hence, the intensity of the acoustic wave incident on the reflector and the intensity of the acoustic wave reflected from the reflector could be assumed to be almost equal. Thus, the acoustic intensity difference caused by the echo signals of both transducers was due to the wave passing through the acoustic matching layer twice. Since the acoustic intensity could be considered proportional to the square of the amplitude of the PZT film vibrating velocity normal to the film direction, and the generated voltage of PZT film could be reasonably assumed to be proportional to the amplitude of PZT film displacement normal to the film direction. Consequently, based on the similar operating frequency (e.g., ∼26 MHz in this study), the acoustic intensity was related to the square of the generated voltage of PZT film.

Comparing the amplitudes of echo signals acquired from both transducers, the ratio of the maximum voltage produced by the transducer with the gradient acoustic matching layer to that without gradient acoustic matching design (18 μm thick Parylene deposition) is approximately 1.33, which indicates the acoustic intensity ratio as 1.78. Meanwhile, the calculated transmission intensity coefficient ratio of the matching layer with micropillar-structured design to the matching layer with an 18 um thick Parylene deposition is approximately 1.38 (Figure 3e based on 26 MHz data points). According to the acoustic reciprocity principle, the resulting intensity transmission ratio of the received signal for the transducer with micro-pillar designed matching layer to that without micropillar design could be calculated as 1.38 × 1.38 = 1.90, which is close to the value of 1.78. Since the acoustic intensity transmission coefficients are derived based on the planar instead of spherical design and some assumptions are made in this discussion, these factors could result in a discrepancy. Due to the relative small difference between the values 1.90 and 1.78 (<7%), the simulated analysis could be reasonably justified by the measured result.

## 5. Conclusions

In this study, a novel pMUT fabrication method capable of producing a better designed acoustic impedance gradient matching layer was proposed, realized, and tested. Although a matching layer equal to one-quarter wavelength of the transmitted wave is an established design principle, its performance for acoustic wave transmission is limited. Based on theoretical study, the acoustic impedance gradient matching layer with a micropillar structure can achieve high acoustic intensity transmission in a wide bandwidth. We successfully fabricated a microstructured PZT pillar array on a large area of spherical PZT film together with Parylene to form an acoustic impedance gradient matching composite. High-quality PZT film deposited on an arbitrary curvature flexible substrate was also demonstrated. The proposed fabrication technique effectively increases the acoustic energy transmission and operation bandwidth of the ultrasonic transducer.

## Figures and Tables

**Table 1.** Physical properties of materials for evaluation.

**Materials**	**Physical Properties**	**Values for Calculation**
PZT	Density	7,500 kg/m^3^
	Possion ratio	0.34
	Young's modulus	136 GPa
	Bulk modulus	142 GPa

Parylene	Density	1,400 kg/m^3^
	Possion ratio	0.4
	Young's modulus	3.88 GPa
	Shear modulus	1.39 GPa
	Bulk modulus	6.47 GPa

Water	Density	1,000 kg/m^3^
	Bulk modulus	2.20 GPa
